# Chimney endovascular aneurysm repair-induced mesenteric ischemia: a report of an extremely rare complication

**DOI:** 10.1016/j.jvscit.2023.101259

**Published:** 2023-07-04

**Authors:** Pierfrancesco Antonio Annuvolo, Federico Pascucci, Fabrizio Minelli, Marco Natola, Tommaso Donati, Yamume Tshomba

**Affiliations:** Unit of Vascular Surgery, Fondazione Policlinico Universitario Gemelli IRCCS – Università Cattolica del Sacro Cuore, Rome, Italy

**Keywords:** Case report, Ch-EVAR, Chimney, EVAR, Mesenteric ischemia

## Abstract

The chimney endovascular aneurysm repair (Ch-EVAR) technique has progressively increased in popularity in the treatment of complex aortic aneurysms. However, the long-term results of this technique still must be assessed, especially in comparison to custom-made solutions. The patency of chimney grafts has always been one of the important issues with the Ch-EVAR technique. However, interactions between nonstented aortic side branches and chimney stent grafts have rarely been discussed. In the present case report, we describe a rare case of mesenteric ischemia due to superior mesenteric artery ostium coverage by the misalignment of a renal stent graft in a Ch-EVAR.

First described by Greenberg et al^1^ in 2003, the chimney endovascular aneurysm repair (Ch-EVAR) technique has progressively increased in popularity for the treatment of complex aortic aneurysms. In the Ch-EVAR technique, off-the-shelf parallel stent grafts are used to create an adequate proximal sealing zone and to maintain, at the same time, perfusion of the visceral arteries.

The ideal combination of abdominal endografts and commercially available stents has been the object of several investigations. Donas et al^2^ were the first to report promising results with a standardized combination of materials. Several clinical and research studies have subsequently contributed to a better understanding of the optimal oversizing and chimney graft choice.^3^

In 2015, the PERICLES (performance of the chimney technique for the treatment of complex aortic pathologies) registry showed promising results for Ch-EVAR, with results comparable to those for other complex endovascular options.^4^ Even at long-term follow-up, the Ch-EVAR technique has been confirmed as a valid, readily available alternative to custom-made solutions, with good clinical and technical results.^5^

The patency of chimney grafts has always been one of the important issues with the Ch-EVAR technique. However, interactions between nonstented side branches and chimney stents have rarely been discussed. A literature analysis was performed by the first two authors (P.A.A., F.P.). From our research, it emerged that superior mesenteric artery ostium coverage is not a previously reported complication of Ch-EVAR with double renal artery chimney grafts.

In the present case report, we describe this potential rare complication that resulted in mesenteric ischemia in a patient treated using the Ch-EVAR technique. This case report has been reported in line with the SCARE (surgical case report) criteria.^6^ The patient provided written informed consent for the report of his case details and imaging studies.

## Case report

A 78-year-old man presented to our institution with a juxta-renal abdominal aortic aneurysm (AAA) measuring 59 mm in maximum diameter as documented by computed tomography angiography (CTA). He had a history of smoking, chronic obstructive pulmonary disease requiring oxygen therapy, parkinsonism, benign prostatic hypertrophy, left inguinal repair, and appendectomy. Considering our patient's medical history, age, and anatomic features, the decision was made to perform EVAR with bilateral renal artery stenting using the chimney technique (Ch-EVAR). The procedure was performed in a hybrid operating room with the patient under local anesthesia.

Aortic catheterization was performed through bilateral percutaneous femoral access. The renal arteries were subsequently catheterized through bilateral percutaneous brachial access. A bifurcated abdominal aortic stent graft was deployed (Endurant IIs 36-14-103 mm; Medtronic Inc), and two balloon-expandable covered stents (BeGraft, 7 × 57 mm; Bentley InnoMed) were placed in the renal arteries. The completion angiogram showed the correct positioning of the endograft, regular patency of both renal arteries and superior mesenteric artery (SMA) with no evidence of endoleaks. The patient recovered well from the procedure without complications and was discharged home on postoperative day 3. The patient did not present for the planned 1-month follow-up CTA.

**Fig 1:**
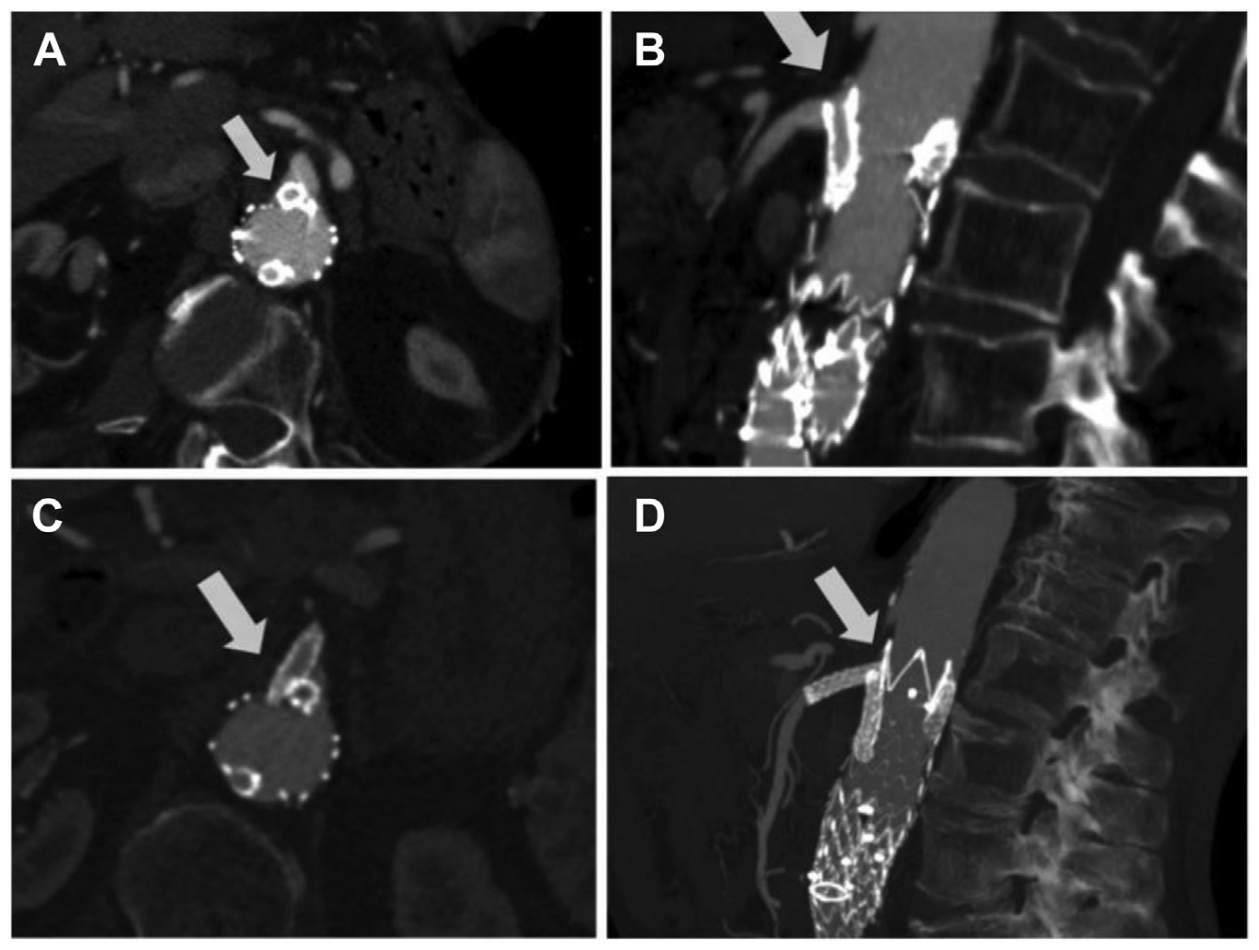
**A,B,** Axial and sagittal preoperative computed tomography angiography (CTA) views showing partial coverage of the superior mesenteric artery (SMA) ostium by the right renal artery (RRA) chimney graft. **C,D,** Axial and sagittal postoperative CTA views showing the stent positioned in the SMA.

At 3 months after the procedure, he presented for our attention with the report of the occurrence of disabling postprandial abdominal pain and significant weight loss. An urgent CTA showed partial coverage of the SMA ostium by the right renal artery (RRA) chimney graft (Fig 1, *A* and *B*). Given the clinical presentation and CTA findings, a decision was made to proceed with an adjunctive procedure of SMA stenting.

With the patient under local anesthesia, bilateral percutaneous brachial access was obtained. The SMA was catheterized from the left brachial access, and the RRA was catheterized from the right brachial access, and a 6F sheath (Flexor; Cook Medical) was advanced over a stiff wire (SMA: Rosen; Cook Medical; RRA: Amplatz super stiff; Boston Scientific) in the target vessels. A 7 × 40-mm balloon (Oceanus 35; iVascular) was positioned in the RRA, and a balloon-expandable covered stent (BeGraft, 7 × 37 mm; Bentley Innomed) was positioned in the SMA. The stent was released, and, simultaneously, the balloon was inflated. The completion angiogram documented the correct positioning of the SMA stent, regular patency of the endoprosthesis, and previously implanted renal stents. The patient was discharged home on postoperative day 2 without any complications. Dual antiplatelet therapy with clopidogrel 75 mg daily and acetylsalicylic acid 100 mg daily was prescribed for 6 months.

**Fig 2:**
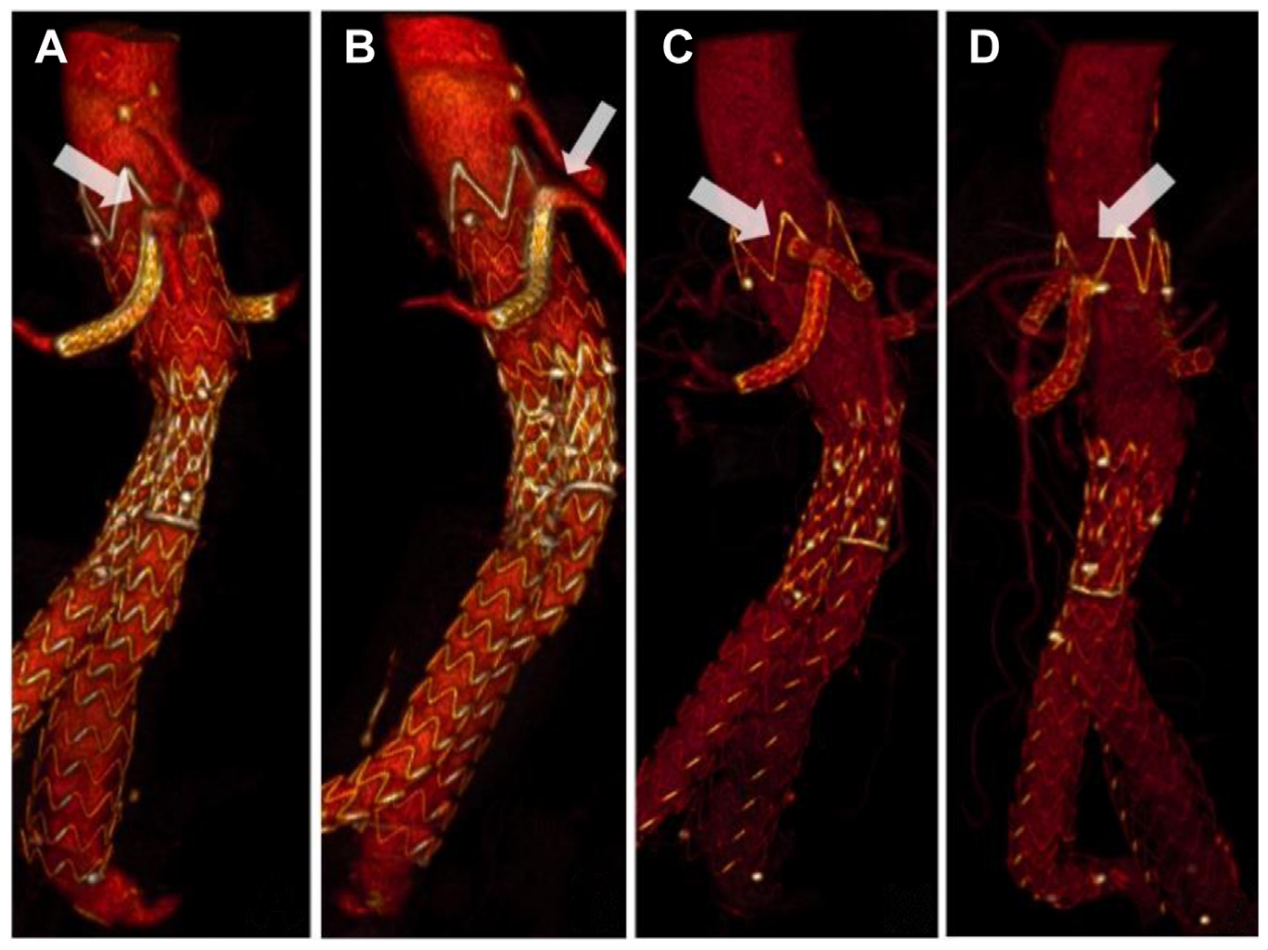
**A,B,** Preoperative three-dimensional reconstruction of computed tomography angiography (CTA). **C,D,** Postoperative three-dimensional reconstructions of CTA.

The 1-month follow-up CTA showed correct positioning of the aortic stent graft, complete exclusion of the aneurysm, no evidence of gutter formation or endoleaks, and regular patency of the SMA and renal arteries (Fig 1, *C* and *D*). Fig 2 shows the preoperative and postoperative CTA three-dimensional reconstructions, demonstrating resolution of the SMA ostium coverage by stenting.

At the 3- and 6-month follow-up visits, the patient was in good clinical condition, with stable renal function and completely resolved postprandial symptoms. An abdominal duplex ultrasound documented the regular patency of the SMA and renal arteries.

## Discussion

The treatment of AAAs extending above the renal arteries or with an unfavorable infrarenal neck for standard EVAR remains challenging, especially in emergency settings.^7^ Although open surgery has remained the treatment of choice for many cases, during the past decades, many endovascular techniques have been developed to offer a valid and less-invasive alternative for patients considered unfit for open repair.

Among these, the Ch-EVAR technique is now a validated procedure that has shown acceptable results in terms of technical success and medium- to long-term outcomes,^8^^,^^9^ even compared with treatments considered more “anatomic” and potentially more stable, such as fenestrated or branched EVAR. However, these techniques are technically demanding for surgeons, with high costs and long manufacturing times.

Physician-modified endografts, originally developed as emergency treatment for aortic arch diseases, could represent an additional alternative to Ch-EVAR, even in the abdominal aorta. However, the techniques have not yet been well-standardized, and particular expertise in graft modification is required.^10^

The main issue regarding the Ch-EVAR technique remains the development of type Ia endoleaks due to the inadequate proximal apposition between the aortic endograft and the chimney graft.^11^ The reported incidence of type Ia endoleaks after Ch-EVAR varies, reaching 35% in some reports at completion angiography. However, it must be highlighted that the spontaneous resolution rate of early gutter-related type Ia endoleaks was estimated to be 44.3%, 65.2%, and 88.4% at 6, 12, and 18 months after the procedure, respectively.^12^

Another relevant issue regarding Ch-EVAR remains late patency and the stability of chimney grafts, materials originally not designed to be used in chimney configuration. However, despite the satisfying data regarding the long-term patency rates of chimney grafts from the PeRfOrmance of The EndurAnt abdominal stent-Graft in the treatment Of paraRenal pAthologieS by the chimney technique (PROTAGORAS) study, stent thrombosis can occur due to compression or kinking. This could be especially common in the case of very closely placed multiple chimney stents.

The anatomic characteristics appear to be extremely relevant for good outcomes using the Ch-EVAR technique. A widely anterior origin of the renal arteries or the proximity of the visceral vessel ostia can cause complications in terms of patency of nonstented vessels, unless all the closest branches are stented. In addition, the more chimneys placed, the greater the increase in the risk of type Ia endoleaks resulting from gutters and intrastent thrombosis due to conflict between closely placed chimneys. In our patient, an accurate evaluation of the preoperative imaging study, a slightly anterior origin of the RRA was detected. This anatomic characteristic probably influenced the SMA ostium coverage in our patient. Even a preoperative evaluation of the angulation of the visceral arteries in three-dimensional space can usually predict how the chimney stents will conform to the patient’s anatomy. To avoid this unpleasant complication, the correct intraoperative orientation of the sheaths and stents can be facilitated by multiple projections in different angulations. In addition, preventive catheterization of the SMA could facilitate eventual stenting of the vessel, if necessary.

## Conclusions

The role of the Ch-EVAR technique in the management of complex AAAs is well established. This technique proved to be extremely useful for extending the proximal sealing zone for juxtarenal aortic aneurysms, especially in emergency settings. However, the long-term outcomes in terms of implant stability and interactions between stent grafts and nonstented visceral arteries remain uncertain. Further studies are needed to better understand the potential conflicts and shifts of parallel grafts used in Ch-EVAR configurations at long-term follow-up. An ad hoc analysis will help clarify the potential issues and identify potential improvements in the technique.
